# Chloroplast genomes of two *Pueraria* DC. species: sequencing, comparative analysis and molecular marker development

**DOI:** 10.1002/2211-5463.13335

**Published:** 2021-12-26

**Authors:** Jishuang Li, Meng Yang, Yanni Li, Mei Jiang, Chang Liu, Meijun He, Bin Wu

**Affiliations:** ^1^ Institute of Medicinal Plant Development Chinese Academy of Medical Sciences and Peking Union Medical College Beijing China; ^2^ Institute of Chinese Medicinal Materials Hubei Academy of Agricultural Sciences Enshi China

**Keywords:** chloroplast genome, comparative analysis, molecular marker, *Pueraria lobata* (Willd.) Ohwi, *Pueraria thomsonii* Benth.

## Abstract

*Puerariae lobatae* radix (Ge‐Gen in Chinese) and *Puerariae thomsonii* radix (Fen‐Ge) are widely used as medicine and health products, particularly in Chinese medicine. Puerarin and daidzein are the primary bioactive compounds in *Puerariae* radix. These isoflavones have been used to treat cardiovascular and cerebrovascular diseases, hypertension, diabetes, and osteoporosis. The content of puerarin in Ge‐Gen is about six times higher than that in Fen‐Ge, so its use has a higher pharmacological effect. It is therefore of great importance to effectively distinguish between these two species. However, because their basal plants, *P*. *lobata* (Willd.) Ohwi and *P*. *thomsonii* Benth., possess an extremely similar appearance, and detecting the level of chemical constituents is just a rough distinction, it is necessary to develop more efficient identification approaches. Here the complete chloroplast genomes of *P*. *lobata* and *P*. *thomsonii* were deciphered, including sequencing, assembly, comparative analysis, and molecular marker development. The results showed that they are 153,393 and 153,442 bp in length, respectively; both contain 124 annotated genes, including eight encoding rRNA, 29 encoding tRNA, and 87 encoding proteins. Phylogenetic analysis showed that they form a clade, indicating that they originate from the same ancestor. After obtaining 10 intergenic/intronic regions with a genetic distance greater than 0.5 cm, primers were designed to amplify regions of high variability in *P*. *lobata* and *P*. *thomsonii*. Finally, a 60‐bp differential base fragment, located in the intron of *rpl16*, was developed as a molecular marker to efficiently distinguish between these two species.

## Graphical abstract

The content of the medicinal isoflavone puerarin in the root of *Puerariae lobatae* (Ge‐Gen) is about six times higher than that in *Puerariae thomsonii* (Fen‐Ge), but these two plants are visually indistinguishable. By deciphering the complete chloroplast genome of these two plants, the authors developed a molecular marker to efficiently distinguish between them.

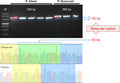

**Abbreviations**
CP: chloroplast
IR: reverse complementary regions
LSC: long single copy region
*P. lobata*
: *Pueraria lobata* (Willd.) Ohwi
*P. thomsonii*
: *Pueraria thomsonii* Benth.
SSC: short single copy region
SSRs: simple sequence repeats

*Puerariae lobatae* radix, known as Ge‐Gen in Chinese, is a popular traditional Chinese herbal medicine that was first described in the Shennong Herbal Classic. The genus *Pueraria* DC. belongs to the Fabaceae family and comprises about 20 species worldwide. It is mainly distributed in East Asian countries, among which China is the distribution center, accounting for 11 species. The dried roots of Ge‐Gen have been widely used for the treatment of influenza, muscle stiffness, and other sicknesses [1]. Owing to a multi‐origin traditional Chinese medicine, there are two *Pueraria* species: *Pueraria lobata* (Willd.) Ohwi and *Pueraria thomsonii* Benth., whose roots were considered the main source of Ge‐Gen because of the wide distribution and its traditional Chinese herbal medicine value, as recorded in the Chinese Pharmacopoeia, 2000 edition [2]. Isoflavones, including puerarin and daidzein, are the primary bioactive compounds in Ge‐Gen. Modern pharmacological studies have proved that puerarin treats cardiovascular and cerebrovascular diseases, hypertension, diabetes, as well as osteoporosis [3], while daidzein can be used in the prevention of high blood alcohol level [4].

According to previous studies, the contents of isoflavones and polysaccharides in *P*. *lobata* are both higher than in *P*. *thomsonii*, especially the main bioactive compounds puerarin and daidzein [5, 6, 7]. This means that the pharmacological effect of *P*. *lobata* is much better than *P*. *thomsonii*. As a result, for accuracy of medication, since 2005 the roots of *P*. *lobata* and *P*. *thomsonii* have been separately used as Ge‐Gen and Fen‐Ge in the Chinese Pharmacopoeia; The Chinese Pharmacopoeia (2010 edition) specifies the lower limit of puerarin in *P*. *thomsonii* and *P*. *lobata* are 0.3% and 2.4%, respectively. However, the morphological characteristics of these two species are too similar to distinguish. They are both perennial woody lianas, up to 8 meters in length. They have thick and massive roots; woody stems at the base; pinnate compound leaves growing with three leaflets; ovate‐shaped accumbent stipules; campanulate calyx, covered with yellowish‐brown pubescent; purple corolla, obovate petals; flat pod, covered with brown bristles. The main difference between these two species is that the leaflets of *P*. *thomsonii* are rhomboid‐ovate or broadly ovate, the apexes are acute or apiculate, the bases are truncate, the lateral leaflets are slightly smaller and more oblique, and the petals are circular. While the shape of dried roots of the two plants is extremely similar, it is more difficult to identify the processed slices. Moreover, their morphological characteristics are susceptible to environmental conditions, which increases the difficulty for identification [8, 9]. Although some common species can be roughly distinguished by chemical composition content, it is not effective to screen *P*. *lobata* and *P*. *thomsonii* and their common counterfeits using minor differences. Universal molecular markers, such as internal transcribed spacer (ITS), *rbcL* and *psbA*, are widely used for identifying species easily and accurately [10, 11, 12], but wild relatives cannot be distinguished efficiently. Because of the lack of an effective approach for distinguishing these two species, they can easily be confused, which could potentially lead to poor therapeutic effects. As a consequence, it is urgent to develop novel molecular markers.

The chloroplast (CP) is an organelle of great importance in green plants, which plays a crucial role in photosynthesis, carbon fixation, translation, and biosynthesis [13]. According to former reports, the length of CP genomes in most angiosperms generally range from 115 to 165 kb; the structure of CP genomes in plants is highly conserved. CP has a circular genome, independent of the nuclear genome and is maternally inherited. The intraspecific sequences of CP genomes are relatively conserved, while some regions are highly variable, which can be utilized as markers for identification of different species [14]; the substitution rate of the CP genome is lower than the nuclear genome, while higher than the mitochondrial genome. As a result, CP genomes show the potential for the distinction among different species. With significant advances in next‐generation sequencing technologies and bioinformatics, CP genomes have been widely used for phylogenetic analysis and molecular marker development in our and other researchers' studies [15, 16, 17, 18]. However, the CP genomes of *P*. *lobata* and *P*. *thomsonii* have not been thoroughly studied. The complete CP genome of *P*. *thomsonii* has been reported [19], and we can use these data for subsequent experimental comparison and verification, and to conduct further analysis for the identification of the two species. Therefore, the comparative analysis and marker development of CP genome of *Pueraria* DC. represented by *P*. *lobata* and *P*. *thomsonii* will have great development prospects.

In our study, the genomes of *P*. *thomsonii* and *P*. *lobata* were sequenced, assembled, and annotated. Afterwards, a comparative genomic analysis between the two CP genomes was conducted with the aim of finding highly variable regions and establish the phylogenetic position of the *Pueraria* DC. in the Fabaceae family. Moreover, a specific molecular marker from the CP genomes of the two *Pueraria* species was developed and verified. In all, our study aimed to ensure safety in the use of medicinal plants, identify species, and conserve wild *Pueraria* species.

## Materials and methods

### Material preparation, DNA extraction, genome sequencing and assembly

Fresh leaf materials of *P*. *lobata* and *P*. *thomsonii* were collected from Sancha Town, Enshi City, Enshi Prefecture, Hubei Province (109.49°E, 30.30°N), China. Samples of *P*. *lobata* were collected from the Beijing Medicinal Plant Garden (116.28°E, 40.04°N) for reference. Leaves were stored at −80 °C after rinsing. Total DNA was extracted using a plant genomic DNA kit (Tiangen Biotech, Beijing, China). DNA quality was assessed using the Nanodrop spectrophotometer 2000 (ThermoFisher Scientific, Waltham, MA, USA), and the integrity was evaluated by 1.0% agarose gel electrophoresis. The sequencing libraries were prepared by using the TruSeq DNA Sample Prep Kit (Illumina, San Diego, CA, USA). DNA was fragmented into ~500 bp long fragments randomly for paired‐end library construction [20]. The library was sequenced on an Illumina HiSeq 3000 instrument. The raw reads obtained were filtered by trimmomatic (v. 0.32) [21]. spades (v. 3.10.1) [22], clc genomics workbench (v. 7), and novoplasty (v. 4.3) [23] were used for the CP genome assembly [24]. The contigs obtained were identified by Gepard [25]. All the identified contigs were assembled using the Seqman module of dnastar (v. 11.0) [26].

### Annotation and comparative analysis

Dual Organellar GenoMe Annotator (DOGMA) [27], the CP Genome Annotation, Visualization, Analysis, and GenBank Submission (CPGAVAS2) [28] and GeSeq [29] were used to annotate the two genomes. Manual corrections on the positions of the start and stop codons and the intron/exon boundaries were performed based on the entries in the plastome database using the Apollo program [30]. The tRNA genes were identified with tRNAscan‐SE [31] and DOGMA. The circular plastome maps were drawn by ogdraw (v. 1.3.1) [32]. The percentage of GC content and the codon usage were analyzed using the programs Cusp and Compseq in emboss (v. 6.3.1) [33]. Comparative analysis of the CP genomes of *P*. *thomsonii* and *P*. *lobata* was performed using the mVISTA program [34] in Shuffle‐LAGAN mode with default parameters [35].

### Long repeats and simple sequence repeats analyses

The size and location of long repeat sequences, including forward, palindromic, reverse, and complement repeats in the CP genomes of two species were identified by setting the parameter of the Hamming Distance to 3 (sequence identity ≥90%) and the Minimal Repeat Size to 30 through reputer [36]. Simple sequence repeats (SSRs) were detected using MISA software with the minimum repeat number set at 8, 4, 4, 3, 3, and 3 for mono‐, bi‐, tri‐, tetra‐, penta‐, and hexa‐nucleotides, respectively [37].

### Phylogenetic analysis

In this study, the phylogenetic tree was conducted using the maximum likelihood (ML) method: a total of 46 CP genomes were used for the phylogenetic analysis, including *P*. *lobata*, *P*. *thomsonii*, and other 42 of Leguminosae, as well as two outgroup species, *Nicotiana tabacum* and *Arabidopsis thaliana*. The CP genome sequences were downloaded from the NCBI GenBank (https://www.ncbi.nlm.nih.gov/) (Table S1). After screening the protein‐coding genes in the CP genomes, 54 common protein‐coding genes (*atpA*, *atpB*, *atpH*, *atpI*, *ccsA*, *cemA*, *clpP*, *matK*, *ndhC*, *ndhE*, *ndhF*, *ndhG*, *ndhH*, *ndhI*, *ndhJ*, *petA*, *petB*, *petD*, *petG*, *petL*, *psaA*, *psaB*, *psaC*, *psaJ*, *psbA*, *psbB*, *psbC*, *psbD*, *psbE*, *psbF*, *psbH*, *psbI*, *psbJ*, *psbK*, *psbM*, *rbcL*, *rpl14*, *rpl16*, *rpl2*, *rpl20*, *rpoA*, *rpoB*, *rpoC1*, *rpoC2*, *rps11*, *rps14*, *rps15*, *rps18*, *rps19*, *rps3*, *rps4*, *rps7*, *rps8*, *ycf3*) were obtained and then globally matched using Alignment using the Fast Fourier Transform (MAFFT) program [38]. Next, using *N*. *tabacum* and *A. thaliana* as outgroup species, phylogenetic relationships were analyzed using the ML method and the Tamura‐Nei model in MEGA6 [39]. The BOOTSTRAP value, initially set to 1000 times, was used to evaluate nodal support of the phylogenetic tree.

### Sequence divergence analysis and molecular marker development

In order to analyze sequence diversity and selective pressure, sequences of 124 annotated genes and 146 intergenic/intronic regions were extracted from the two CP genomes, and then aligned by the Clustalw2 (v. 2.0.12) program [40] with the options: “‐type=dna, ‐gapopen=10, ‐gapext=2”, pairwise distance were determined with the Distmat program that was implemented in EMBOSS [33] using the Kimura 2‐parameters (K2p) evolution model.

The molecular marker was selected based on the alignment and comparison of mVISTA similarities and divergence analysis results. To amplify these regions, primers were designed using Primer Premier 5 [41]. In order to further confirm whether the molecular marker developed in this study was reliable for the classification and identification of *P*. *lobata* and *P*. *thomsonii*, plant samples of *P*. *lobata* and *P*. *thomsonii* were collected from some other regions (Table S2). The procedure of DNA extraction was as described above. The developed molecular marker was used to amplify the total DNA of *P*. *lobata* and *P*. *thomsonii* from different regions. PCR was conducted with the following program: initial denaturation at 95 °C for 3 min; followed by 35 cycles of amplification at 94 °C for 30 s, 56 °C for 30 s, and 72 °C for 1 min; and final extension at 72 °C for 5 min. Then the PCR products were separated with 1.0% (w/v) agarose gel for 20 min at 120 volts. Finally, the products were sent to the company for sequencing. The primers are listed in Table 2.

## Results

### General features of the CP genomes

**Fig. 1:**
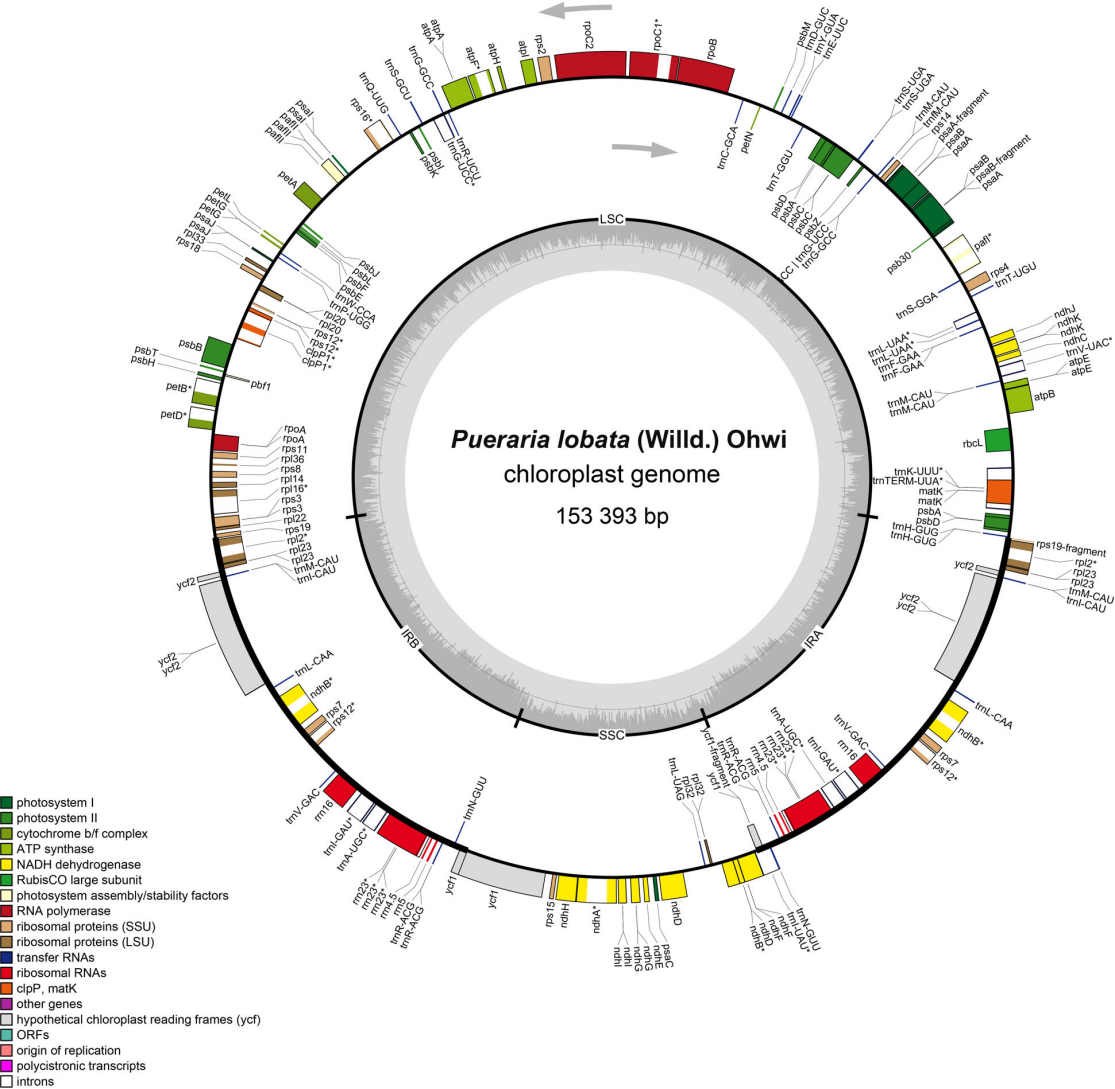
Circular gene map of the complete CP genome of *Pueraria* *lobata*. Genes drawn inside the circle are transcribed clockwise, and those on the outside are transcribed counterclockwise. The dark gray area in the inner circle corresponds to the GC content, whereas the light gray refers to AT content. LSC, large single copy region; SSC, small single copy region; IRa, IRb, copies of inverted repeat regions.

After assembly and annotation, the CP genomes of *P*. *lobata* and *P*. *thomsonii* both display circular molecules with the typical quadripartite structure, including a long single copy region (LSC), a short single copy region (SSC), and a pair of reverse complementary regions (IRa and IRb). The CP genome of *P*. *lobata* from Hubei Province was 153,393 bp in length; while that of *P*. *thomsonii* was 153,442 bp; both of the total GC contents were 35.41% (Fig. 1, Table 1, and Fig. S1).

**Table 1:** Base composition in the CP genomes of *Pueraria* *lobata* and *Puerariae* *thomsonii*.

Chinese name	Latin name	Length (bp)				GC content (%)			
		Total	LSC	SSC	IR	Total	LSC	SSC	IR
Enye	*P. lobata*	153,393	84,089	17,992	25,656	35.41%	32.86%	28.90%	41.87%
Guangfen	*P. thomsonii*	153,442	84,163	17,998	25,640	35.41%	32.87%	28.90%	41.88%

Both *P*. *lobata* and *P*. *thomsonii* have 124 annotated genes: 87 protein‐coding genes, 29 tRNA genes, and eight rRNA genes. The 87 protein‐coding genes could be divided into three main types: the first one refers to a total of 29 self‐replicated genes; the second one refers to a total of 46 genes related to photosynthesis; the third one refers to a total of 12 other protein‐coding genes (Table S3).

There were 58 protein‐coding genes and 19 tRNA genes in the LSC region of *P*. *lobata*, accounting for 53.38% of the total length of LSC; there were five protein‐coding genes, four rRNA genes, and eight tRNA genes in each IR region, accounting for 67.80% of the total length of IR; and there were 12 protein‐coding genes and one tRNA gene in the SSC region, accounting for 50.48% of the total length of the SSC. The total gene sequence length of each region accounted for 58.57% of the CP genome length of *P*. *lobata*, as (Table S4). There were 61 protein‐coding genes and 21 RNA genes in the LSC region of *P*. *thomsonii*, accounting for 52.82% of the whole sequence of LSC; there were seven protein‐coding genes and 11 RNA genes in both IR regions, accounting for 65.68% of the total length of IR; and there were 13 protein‐coding genes and one tRNA in the SSC region, accounting for 50.46% of the total length of the SSC. The total encoding gene sequence accounted for 56.34% of the total length of the CP genome of *P*. *thomsonii*.

**Fig. 2:**
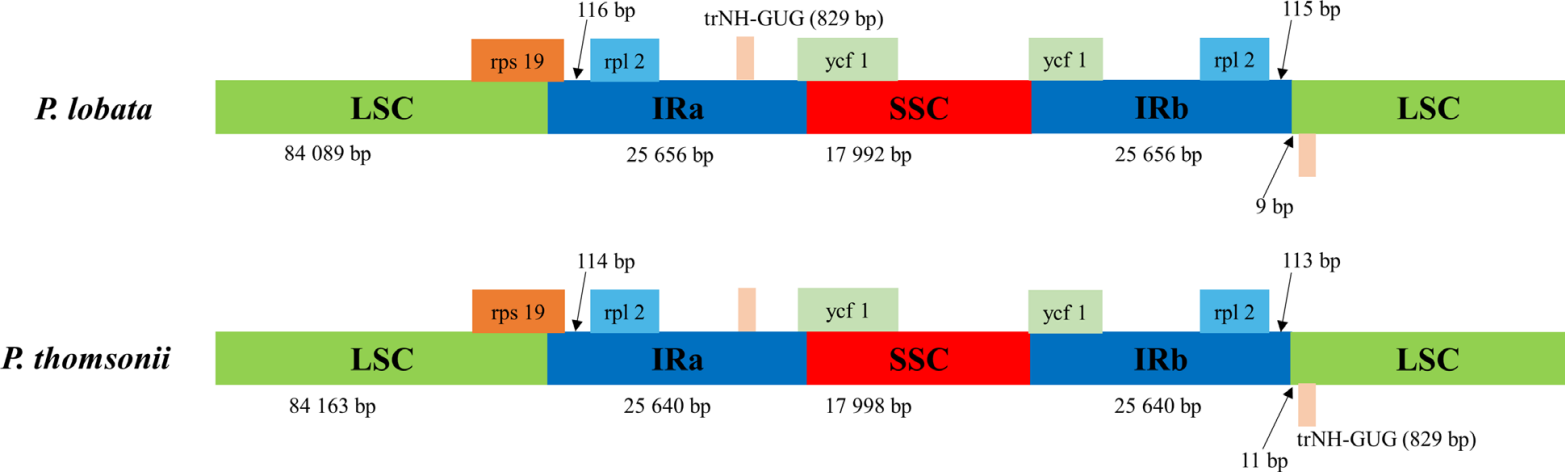
Comparison of boundary distance among adjacent genes and junctions of the LSC, SSC, and IRs regions between CP genomes of *Pueraria* *lobata* and *Puerariae* *thomsonii*. The figure is not to scale with respect to sequence length.

Additionally, the four CP boundaries (LSC‐IRA, IR‐SSC, SSC‐IRB, IRB‐LSC) and the adjacent genes of the CP genomes of *P*. *lobata* and *P*. *thomsonii* were compared in detail (Fig. 2). Genes of *P*. *lobata* and *P*. *thomsonii* were basically the same at the junction of each region, among which the *rps19* gene stretched across LSC and IRa; *ycf1* stretched across IRa and SSC; the distance between *rps19*, *rpl2* and the boundary of *P*. *lobata* was two bp longer than that of *P*. *thomsonii*, respectively; *trNH‐GUG* was at the head of the LSC region and it is nine bp away from IRb in *P*. *lobata*, while it is 11 bp away from IRb in *P*. *thomsonii*.

There were 22 genes with introns in *P*. *lobata*, among which 20 genes had a single intron, two genes had two introns; while in *P*. *thomsonii* there were 22 genes with introns, among which 20 genes had one intron, and two genes had two introns (Tables S5 and S6).

### Most discrepancies exist in intergenic/intronic regions

**Fig. 3:**
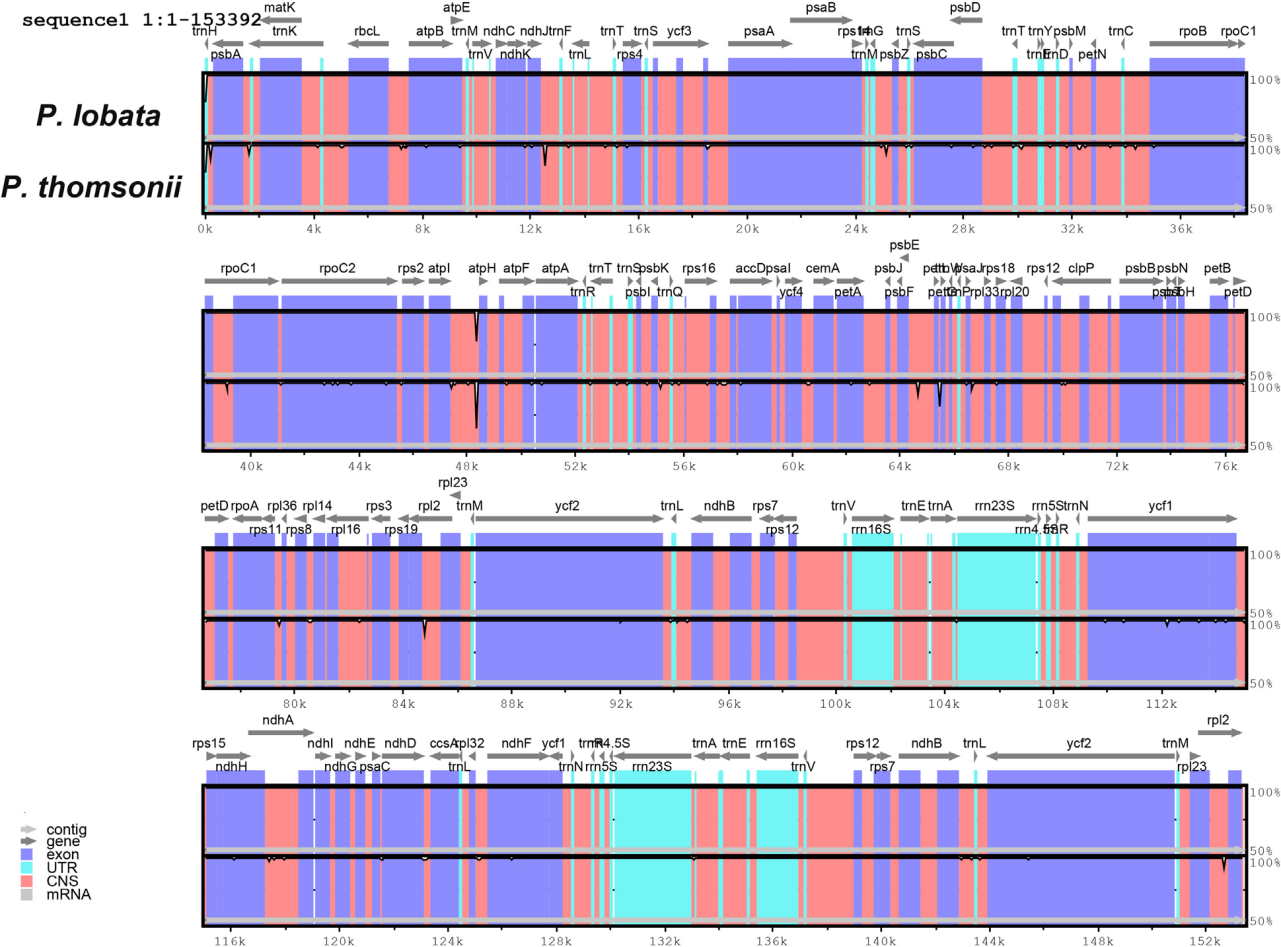
Structure comparison of the complete CP genomes of *Pueraria* *lobata* and *Puerariae* *thomsonii* using the mVISTA program. Equal amounts of total DNA from three individuals of each specie were pooled and sequenced. The gray arrows and bold lines at the top of the global alignment represent gene orientation and IRs location, respectively. The *y* axis represents a 50–100% of identity.

As shown in Fig. 3, although the CP genomes of the two species had the same overall structure and similar regions, there were some nucleotide polymorphisms, and these differences were mostly concentrated in intergenic/intronic regions. Additionally, fewer differences within protein‐coding regions could also be found.

Then, sequences of 124 annotated genes and 146 intergenic/intronic regions were extracted from the CP genomes of *P*. *lobata* and *P*. *thomsonii* for genetic distance analysis. The result showed that the degree of variation of the protein‐coding region was not considerable at all, and the genetic distance of the intergenic/intronic region was slightly larger than that of the protein‐coding region. The result was consistent with the result of the mVISTA global comparison and the result of comparative analysis among the CP genomes of other species that had been published [42]. The genetic distance of the protein‐coding region ranged from 0.00 to 0.25, while that of the intergenic/intronic region was from 0.00 to 0.97. After screening, we obtained 10 protein‐coding regions with genetic distance >0.1, and 10 intergenic/intronic regions with genetic distance >0.5 (Tables S7 and S8). The corresponding sequence information was extracted from the CP genome sequences of *P*. *lobata* and *P*. *thomsonii*. Furthermore, the molecular marker can be subsequently developed.

### *Pueraria lobata* possesses more repeat sequences

**Fig. 4:**
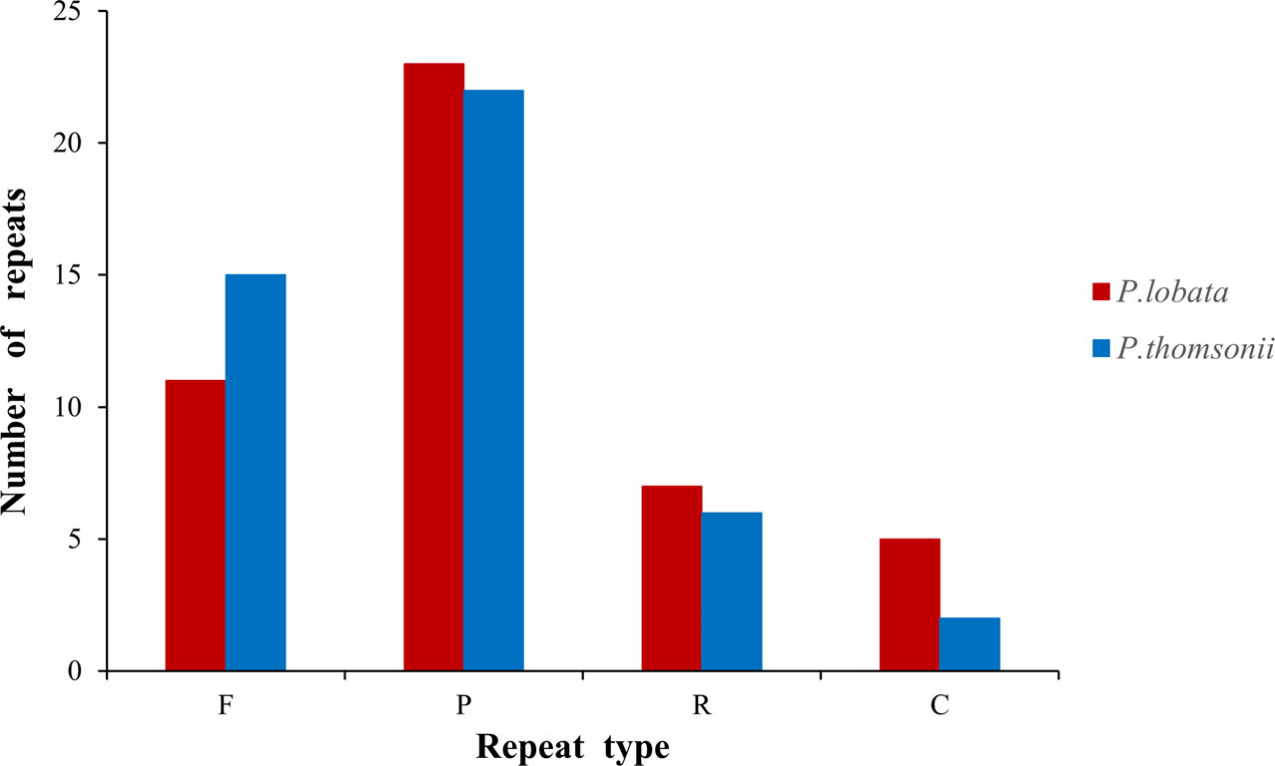
Repeat sequence statistical analysis of the CP genomes of *Pueraria* *lobata* and *Puerariae* *thomsonii*. Equal amounts of total DNA from three individuals of each specie were pooled and sequenced. “F” refers to forward repeat, “P” refers to palindromic repeat, “R” refers to reverse repeat, and “C” refers to complementary repeat.

Repeat units play important roles in genome evolution, and they are distributed quite frequently in CP genomes [43, 44, 45]. The repeat sequence statistics of *P*. *lobata* and *P*. *thomsonii* in this study are shown in Fig. 4. In the CP genome of *P*. *lobata*, a total of 46 repeat sequences with length of at least 30 bp and similarity of at least 90% were found. Among them, there were 23 palindromic repeat sequences, 11 forward repeats, seven reverse repeats, and five complementary repeats; in the CP genome of *P*. *thomsonii*, a total of 45 repeat sequences with length of at least 30 bp and similarity of at least 90% were found. Among them, there were 22 palindromic repeats, 15 forward repeats, six reverse repeats, and two complementary repeats. Generally speaking, the forward repeat sequences and the palindromic ones are the most abundant in both species. This result is consistent with the analyses of repeat sequences of other angiosperm CP genome sequences [46].

The difference of the total number of repeats was not obvious, while the main difference of number was reflected in the forward repeat sequences and the complementary ones. The forward repeat sequences in the CP of *P*. *lobata* were four fewer than those in *P*. *thomsonii*, while the complementary ones in the CP of *P*. *lobata* were three more than those in *P*. *thomsonii*.

The SSRs analysis results are shown in Table S9. A total of 284 SSRs were detected in *P*. *lobata*, while 283 SSRs were detected in *P*. *thomsonii*. Apart from the one more dinucleotide repeat sequence AT/AT in *P*. *lobata* than in *P*. *thomsonii*, the number of other tandem repeats in both species was similar. Among the detected repeat sequences, there were 182 A/T single nucleotide repeat sequences, which were the most abundant, and accounted for 64.1% of the total number of repeat sequences; both had three G/C single nucleotide repeats. There were 62 and 63 AT/AT dinucleotide repeat sequences in total, respectively; both had 18 AG/CT dinucleotide repeats. Only one trinucleotide repeat sequence AAG/CTT was detected. The types of tetranucleotide repeat sequences included one AATC/ATTG repeat, two AATT/AATT repeats, three AAAT/ATTT repeats, and 12 AGAT/ATCT repeats (Tables S10 and S11).

### The close phylogenetic relationship

**Fig. 5:**
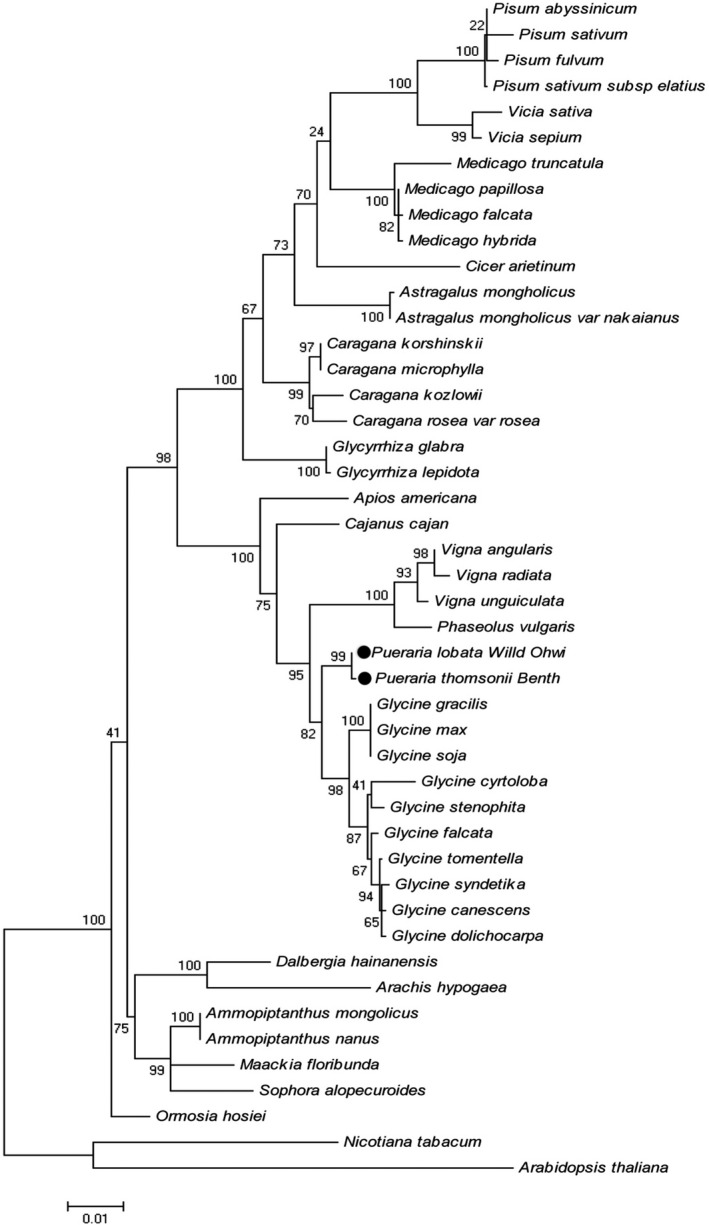
Phylogenetic tree of *Pueraria* *lobata*, *Puerariae* *thomsonii,* and the other 42 species of legumes based on the shared gene sequences among the CP genomes. The numbers on each branch represent bootstrap support.

The ML phylogenetic tree construction results are shown in Fig. 5. It shows that the phylogenetic positions of the two species are basically consistent with the known evolutionary relationship of species. In the whole phylogenetic tree, *Ormosia* is a single branch, while the other species are mainly divided into two branches. *Arachis hypogaea*, *Maackia* *floribunda*, *Sophora alopecuroides*, *Ammopiptanthus hainanensis,* and *Dalbergia hainanensis* are closely related, so they were classified as one clade; most of the remaining species belong to the other main clade, including *Glycyrrhiza glabra*, *Medicago falcata*, *Astragalus mongholicus,* and *Caragana*. *Pueraria* *lobata*, *P*. *thomsonii, Glycine max*, *Vigna unguiculata,* and *Caragana* were classified into another subordinate clade. *Pueraria* *lobata* and *P*. *thomsonii* belong to the same small clade.

### Identification marker for distinguishing *P*. *lobata* from *P*. *thomsonii*

Through the comparative analysis of genomes and the genetic distance analysis results, the target sequences from the high variability regions were extracted for designing primers and screening out the resulting molecular markers. The *P*. *lobata* and *P*. *thomsonii* samples from Enshi, Hubei province, and the Beijing Medicinal Plant Garden were tested by PCR amplification, gel electrophoresis, and PCR product sequencing and verification. Aiming at the indels that existed among the amplified fragments of *P*. *lobata* and *P*. *thomsonii*, a pair of primer sequences named YF‐60 were tested, which means that the 60‐bp base difference could be used as the molecular marker to distinguish *P*. *lobata* from *P*. *thomsonii* (Table 2).

**Table 2:** The specific molecular marker primer.

Primer name	Primer sequence	PCR products size (bp)	
		*Pueraria* *lobata*	*Puerariae thomsonii*
YF‐60	Forward: CTAGATAATCCGAAGCGATGC	338	398
	Reverse: TCAGAGAAGGTAGGGTTCCTC		

**Fig. 6:**
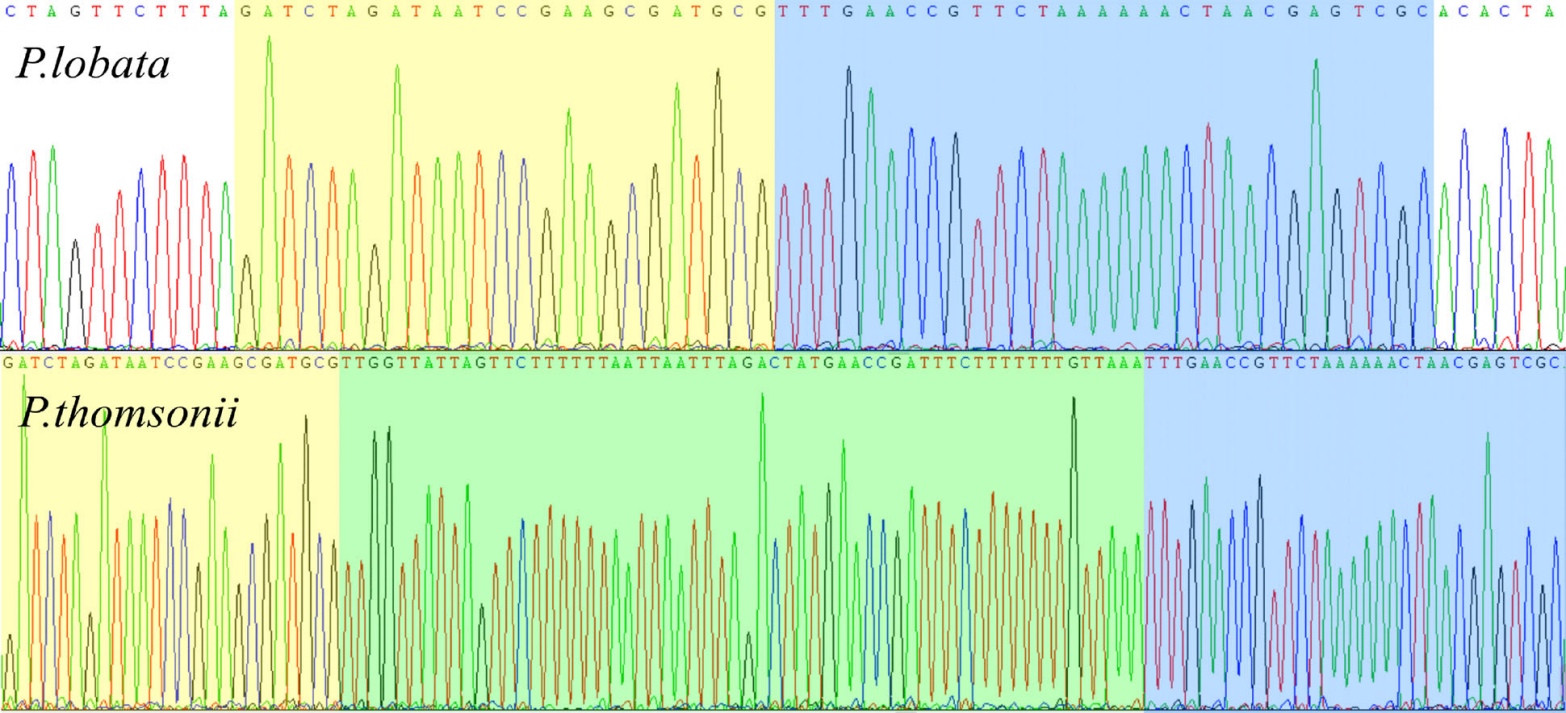
Primer YF‐60 amplification chromatogram. Equal amounts of total DNA from three individuals of each specie were pooled and sequenced. The blue and yellow background represent the common sequence of *Pueraria* *lobata* and *Puerariae* *thomsonii*, while the green background represents the unique sequence of *P*. *thomsonii*.

**Fig. 7:**
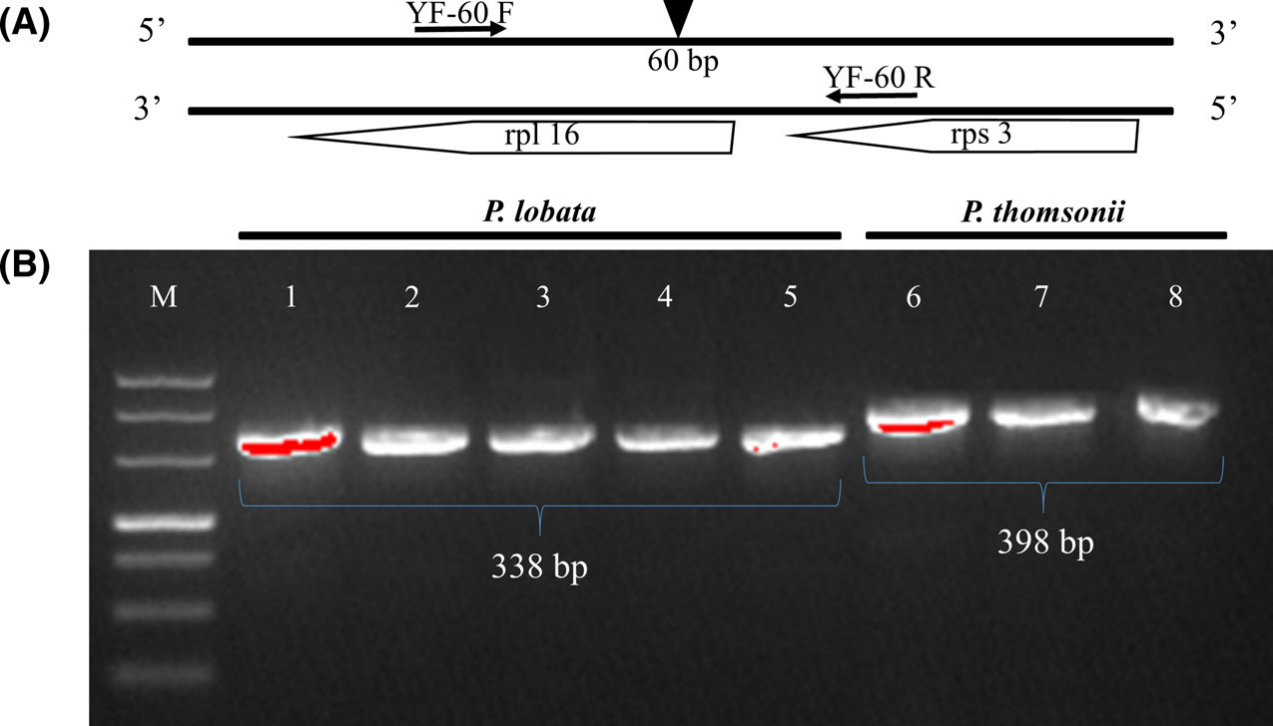
(A) The target DNA double‐strand fragment extracted from the highly variable region. YF‐60 represents primer; 60 bp represents the differential base fragment; rpl16 represents the gene where YF‐60 F and the 60‐bp differential base fragment are located; rps3 represents the gene where YF‐60 R is located. (B) PCR amplification of *Pueraria* *lobata* and *Puerariae* *thomsonii* from different habitats using YF‐60 in 1% agar‐gel electrophoresis were 338 bp and 398 bp, respectively. “M” represents DL500 ladder; lanes 1–5 represent *P*. *lobata* from Hubei, Hubei, Beijing, Guizhou, and Liaoning, respectively; while lanes 6–8 represent *P*. *thomsonii* from Guangxi, Hunan, and Hubei, respectively.

This pair of high‐resolution primers could clearly distinguish the *P*. *lobata* sample from the *P*. *thomsonii* sample with the highest degree of differentiation. Comparison of YF‐60 amplified sequences showed that there was a lack of a 60‐bp fragment in *P*. *lobata* compared with *P*. *thomsonii*. Sanger chromatograms are shown in Fig. 6. Gene *rpl16* was chosen as the specific molecular marker region with high divergence, where the 60‐bp differential base fragment as well as YF‐60 F exists; while gene *rps3* is the area where YF‐60 R locates. Specifically speaking, the 60‐bp differential base fragment locates in the intronic region (Fig. 7A).

After PCR amplification of DNA extracted from the *P*. *lobata* and *P*. *thomsonii* samples collected from the seven different regions using primer YF‐60, a 1% agarose gel electrophoresis was conducted for the amplified products (Fig. 7B). The result verified the effectiveness of our developed molecular marker: *P*. *lobata* could be clearly identified from *P*. *thomsonii* through it.

## Discussion

The CP genomes of *P*. *lobata* and *P*. *thomsonii* were assembled and the general features of the two CP genomes were analyzed. The analyses helped us to find that the CP genomes of *P*. *lobata* and *P*. *thomsonii* both display single circular molecules with the typical quadripartite structure. The length, region composition, and GC content of different specimens of *P*. *lobata* from Hubei were essentially identical. There was almost no difference among different specimens of *P*. *thomsonii* from Hubei. However, there existed a few differences between *P*. *lobata* samples for reference collected from Beijing and those *P*. *lobata* control samples from Hubei, which may provide some references and basis for the identification of genuine medicinal materials in the future. According to the analyses of the total length of CP and the length of each region, the differences of length between the two species mainly existed in the LSC and IR regions, while the SSC was similar in size. Moreover, the composition and structure of the CP genomes of *P*. *lobata* and *P*. *thomsonii* are similar to that of most angiosperms, especially legumes, indicating that the CP genome has a high degree of genetic conservation. As a result, it provided a basis for phylogenetic analysis and the development of a molecular marker.

The Gepard comparison result just showed that there were no obvious discrepancies in structure and length between them, but mVISTA presented a more detailed version of the contrast. Through comparative analysis we found a few distinguishable sites; most of the discrepancies existed in the intergenic/intronic regions, and the corresponding sequence information was extracted from the CP genome sequences of *P*. *lobata* and *P*. *thomsonii*.

Through the statistical analysis of repeat sequences, it was found that the number of repeat sequences in the CP genome of *P*. *lobata* was higher than in *P*. *thomsonii*. The primary repeat sequence difference was reflected in the forward and complementary repeats. In general, the forward and palindromic repeats were most abundant and the result was consistent with other angiosperm CP genome sequences analyses [46]. In addition, most of the SSRs were located in intergenic/intronic regions. Among the detected repeat sequences, the A/T single nucleotide repeat sequences were the most common overall, and there were many tetranucleotide repeat sequences, while pentanucleotide and hexanucleotide repeats were not found.

The chloroplast genome is an important genome resource for accurate phylogenetic reconstruction and classification among related angiosperms [47]. Complete CP genome and protein‐coding gene have been commonly used to confirm phylogenetic relationships at nearly every taxonomic level [48]. In this study, the phylogenetic tree constructed with common genes for the 46 CP genomes showed that the bootstrap values in the legume clade were relatively high, indicating that CP genome sequences could be used for phylogenetic analysis and classification of intergeneric and interspecific relationships in the Fabaceae. With our method, the positions of *P*. *lobata* and *P*. *thomsonii* were verified: both were closely related, forming a single clade, which is consistent with the conclusion of the former *Pueraria* botanical classification research [49].

Through the global comparative analysis and the genetic distance analysis, the diversity and variety of the CP genomes of *P*. *lobata* and *P*. *thomsonii* were revealed; the hypervariable sites were mostly distributed in the intergenic/intronic regions. We identified a 60‐bp differential base fragment between sequences of *P*. *lobata* and *P*. *thomsonii* to be used as a molecular marker, located in the intronic region of gene *rpl16*. Finally, a pair of efficient primers was developed to distinguish these two species. *Rpl16* is a protein‐coding gene, and the coded protein is named 50S ribosomal protein L16 (QGT34303.1). According to previous studies, the abundance of RPL16 increased in tolerant or sensitive seedlings under dehydration; [50]; the peptidyltransferase activity of RPL16 was inactivated by a photosensitizer [51]. Consequently, *rpl16* is very likely related to the dehydration response, as well as photosensitivity, to some extent. As for the intron, it was reported that most of the genes expressed at higher levels tend to possess shorter introns. Natural selection has driven introns to smaller sizes in highly expressed genes to reduce the cost of transcription, and small introns may have evolved to fit more genes into smaller regions [52, 53]. Thus, shorter introns in *P*. *lobata* may be an ancestral characteristic of the highly expressed gene *rpl16*, intron length in *rpl16* may have decreased as evolved. Taken together, it is quite possible that *P*. *lobata* has stronger dehydration resistance and photosensitive activity than *P*. *thomsonii*.

For the sake of further confirmation of the accuracy, with the total genomic DNA extracted from leaves of *P*. *lobata* and *P*. *thomsonii* collected from different regions as reference, the YF‐60 marker developed in this study was verified by PCR amplification and the 1% agarose gel electrophoresis. The novel molecular marker developed in this study could effectively distinguish the two *Pueraria* DC. species from different geographical regions. In addition, apart from the published *P*. *thomsonii* CP genome 19, we noticed another released *P*. *lobata* CP genome in NCBI when conducting nucleotide blast (https://www.ncbi.nlm.nih.gov/nucleotide/MT818508.1). Therefore, we further compared and verified the CP genome of *P*. *lobata*, and found no difference from *P*. *thomsonii* in the YF‐60 marker region where it should have. This result just indicated the phenomenon that *P*. *thomsonii* is easily mistaken as *P*. *lobata*, and further supported the availability and application value of our developed molecular marker YF‐60. In all, the developed molecular marker in this study will be beneficial to lay a theoretical foundation for identification of species of *Pueraria* DC. and promote available utilization and protection of wild *Pueraria* DC. resources.

## Conflict of interest

The authors declare no conflicts of interest.

## Author contributions

BW, MH, and CL conceptualized the project. MH and BW administrated the project. JL and MY did the formal analyzing. JL, MY and MJ analyzed and interpreted the data. MY and JL applied the software. JL and MY wrote the original draft. BW supervised the work. JL validated the work. JL, MH, YL, and BW reviewed and edited the article. All authors read and agreed on the published version of the article.

## Supporting information


**Fig. S1.** Circular Gene map of the complete CP genome of *Puerariae* *thomsonii*. Genes drawn inside the circle are the transcribed clockwise, and those on the outside are transcribed counter‐clockwise. The dark gray area in the inner circle corresponds to the GC content, whereas the light gray refers to AT content. LSC: large single copy region, SSC: small single copy region, IRa, IRb: copies of inverted repeat regions.

**Table S1.** CP genome information for phylogenetic tree construction.

**Table S2.** Sample information.

**Table S3.** Annotated genes of CP genome.

**Table S4.** Different genes in different regions in *Pueraria lobata*.

**Table S5.** The lengths of introns for the splitting genes in *Pueraria* *lobata*.

**Table S6.** The lengths of introns for the splitting genes in *Puerariae* *thomsonii*.

**Table S7.** k2p genetic distance of protein‐coding region of *Pueraria* *lobata* and *Puerariae* *thomsonii* ≥ 0.1.

**Table S8.** k2p genetic distance of intergenic/intronic region of *Pueraria* *lobata* and *Puerariae* *thomsonii* ≥ 0.5.

**Table S9.** SSR statistics of CP genome of *Pueraria* *lobata* and *Puerariae* *thomsonii*.

**Table S10.** SSR distribution in *Pueraria* *lobata* CP genome.

**Table S11.** SSR distribution in *Puerariae* *thomsonii* CP genome.

## Data Availability

The CP genomic sequences of *P*. *lobata* and *P*. *thomsonii* were submitted to GenBank and the IDs MZ901204 and MZ901205 were acquired, respectively. The raw data of DNA sequencing for *P*. *lobata* and *P*. *thomsonii* were deposited in the NCBI short read archive (SRA) under accession numbers SRR15508061 and SRR15508060, respectively.

## References

[1] Commission CP . Pharmacopoeia of the People's Republic of China. The Chemical Industry Publishing House; 2020.

[2] Song W , Li YJ , Qiao X , Qian Y , Ye M . Chemistry of the Chinese herbal medicine Puerariae Radix (Ge‐Gen): a review. J Chin Pharm Sci. 2014;23:347–60.

[3] Wei SY , Chen Y , Xu XY . Progress on the pharmacological research of puerarin: a review. Chin J Nat Med. 2014;12:407–14. 24969520 10.1016/S1875-5364(14)60064-9

[4] Lowe ED , Gao G‐Y , Johnson LN , Keung WM . Structure of daidzin, a naturally occurring anti‐alcohol‐addiction agent, in complex with human mitochondrial aldehyde dehydrogenase. J Med Chem. 2008;51:4482–7. 18613661 10.1021/jm800488j

[5] Du G , Zhao HY , Zhang QW , Li GH , Yang FQ , Wang Y , et al. A rapid method for simultaneous determination of 14 phenolic compounds in Radix Puerariae using microwave‐assisted extraction and ultra high performance liquid chromatography coupled with diode array detection and time‐of‐flight mass spectrometry. J Chromatogr A. 2010;1217:705–14. 20036771 10.1016/j.chroma.2009.12.017

[6] Li J , Li C , Gou J , Zhang Y . Molecular cloning and functional characterization of a novel isoflavone 3'‐O‐methyltransferase from *Pueraria lobata* . Front Plant Sci. 2016;7:793. 27458460 10.3389/fpls.2016.00793 PMC4937802

[7] Wang X , Fan R , Li J , Li C , Zhang Y . Molecular cloning and functional characterization of a novel (Iso)flavone 4',7‐O‐diglucoside glucosyltransferase from *Pueraria lobata* . Front Plant Sci. 2016;7:387. 27066037 10.3389/fpls.2016.00387 PMC4814453

[8] Jiang XH , Liu LK , She CW . Study on classification consistency based on morphology and rDNA ITS sequences of Pueraria species. Hubei Agric Sci. 2016;55:940–42.

[9] Reddy CK , Fei L , Xu B . Morphology, crystallinity, pasting, thermal and quality characteristics of starches from adzuki bean (*Vigna angularis* L.) and edible kudzu (*Pueraria thomsonii* Benth). Int J Biol Macromol. 2017;105(Pt:1):354–62. 28705501 10.1016/j.ijbiomac.2017.07.052

[10] Liu C , Liang D , Gao T , Pang X , Song J , Yao H , et al. PTIGS‐IdIt, a system for species identification by DNA sequences of the psbA‐trnH intergenic spacer region. BMC Bioinformatics. 2011;12(Suppl 13):S4. 10.1186/1471-2105-12-S13-S4 PMC3278844 22373238

[11] Trobajo R , Mann DG , Clavero E , Evans KM , Vanormelingen P , McGregor RC . The use of partialcox1, rbcL and LSU rDNA sequences for phylogenetics and species identification within theNitzschia paleaspecies complex (Bacillariophyceae). Eur J Phycol. 2010;45:413–25.

[12] Turenne CY , Sanche SE , Hoban DJ , Karlowsky JA , Kabani AM . Rapid identification of fungi by using the ITS2 genetic region and an automated fluorescent capillary electrophoresis system. J Clin Microbiol. 1999;37:1846–51. 10325335 10.1128/jcm.37.6.1846-1851.1999 PMC84966

[13] Jansen RK , Ruhlman TA . Plastid genomes of seed plants. Genomics Chloroplasts Mitochondria. 2012;103–26.

[14] Parks M , Cronn R , Liston A . Increasing phylogenetic resolution at low taxonomic levels using massively parallel sequencing of chloroplast genomes. BMC Biol. 2009;7:84. 19954512 10.1186/1741-7007-7-84 PMC2793254

[15] Daniell H , Lin CS , Yu M , Chang WJ . Chloroplast genomes: diversity, evolution, and applications in genetic engineering. Genome Biol. 2016;17:134. 27339192 10.1186/s13059-016-1004-2 PMC4918201

[16] Biju VC , Shidhi S , Vijayan S , Rajan VS , Sasi A , Janardhanan A , et al. The complete chloroplast genome of *Trichopus zeylanicus*, and phylogenetic analysis with dioscoreales. Plant Genome. 2019;12:190032. 10.3835/plantgenome2019.04.0032 PMC12810117 33016590

[17] Ye X , Hu D , Guo Y , Sun R . Complete chloroplast genome of *Castanopsis sclerophylla* (Lindl.) Schott: genome structure and comparative and phylogenetic analysis. PLOS ONE. 2019;14:e0212325. 31361757 10.1371/journal.pone.0212325 PMC6667119

[18] Shi H , Yang M , Mo C , Xie W , Liu C , Wu B , et al. Complete chloroplast genomes of two Siraitia Merrill species: comparative analysis, positive selection and novel molecular marker development. PLoS One. 2019;14:e0226865. 31860647 10.1371/journal.pone.0226865 PMC6924677

[19] Miao XR , Niu JQ , Wang AQ , Wang DB , Fan J . Complete chloroplast genome sequence of *Pueraria thomsonii*, an important traditional Chinese medicine plant. Mitochondrial DNA Part B. 2019;4:4163–5. 33366364 10.1080/23802359.2019.1693301 PMC7707730

[20] Stegemann S , Bock R . Experimental reconstruction of functional gene transfer from the tobacco plastid genome to the nucleus. Plant Cell. 2006;18:2869–78. 17085684 10.1105/tpc.106.046466 PMC1693929

[21] Bolger AM , Lohse M , Usadel B . Trimmomatic: a flexible trimmer for Illumina sequence data. Bioinformatics. 2014;30:2114–20. 24695404 10.1093/bioinformatics/btu170 PMC4103590

[22] Bankevich A , Nurk S , Antipov D , Gurevich AA , Dvorkin M , Kulikov AS , et al. SPAdes: a new genome assembly algorithm and its applications to single‐cell sequencing. J Comput Biol. 2012;19:455–77. 22506599 10.1089/cmb.2012.0021 PMC3342519

[23] Nicolas D , Patrick M , Guillaume S . NOVOPlasty: de novo assembly of organelle genomes from whole genome data. Nucleic Acids Res. 2017;45:e18. 28204566 10.1093/nar/gkw955 PMC5389512

[24] Dierckxsens N , Mardulyn P , Smits G . NOVOPlasty: de novo assembly of organelle genomes from whole genome data. Nucleic Acids Res. 2017;45:e18. 28204566 10.1093/nar/gkw955 PMC5389512

[25] Krumsiek J , Arnold R , Rattei T . Gepard: a rapid and sensitive tool for creating dotplots on genome scale. Bioinformatics. 2007;23:1026–8. 17309896 10.1093/bioinformatics/btm039

[26] Sharp PM , Li WH . The codon Adaptation Index–a measure of directional synonymous codon usage bias, and its potential applications. Nucleic Acids Res. 1987;15:1281–95. 3547335 10.1093/nar/15.3.1281 PMC340524

[27] Wyman SK , Jansen RK , Boore JL . Automatic annotation of organellar genomes with DOGMA. Bioinformatics. 2004;20:3252–5. 15180927 10.1093/bioinformatics/bth352

[28] Liu C , Shi L , Zhu Y , Chen H , Zhang J , Lin X , et al. CpGAVAS, an integrated web server for the annotation, visualization, analysis, and GenBank submission of completely sequenced chloroplast genome sequences. BMC Genom. 2012;13:715. 10.1186/1471-2164-13-715 PMC3543216 23256920

[29] Tillich M , Lehwark P , Pellizzer T , Ulbricht‐Jones ES , Fischer A , Bock R , et al. GeSeq – versatile and accurate annotation of organelle genomes. Nucleic Acids Res. 2017;45:W6–11. 28486635 10.1093/nar/gkx391 PMC5570176

[30] Lee E , Harris N , Gibson M , Chetty R , Lewiss S . Apollo: a community resource for genome annotation editing. Bioinformatics. 2009;25:1836–7. 19439563 10.1093/bioinformatics/btp314 PMC2705230

[31] Lowe TM , Eddy SR . tRNAscan‐SE: a program for improved detection of transfer RNA genes in genomic sequence. Nucleic Acids Res. 1997;25:955–64. 9023104 10.1093/nar/25.5.955 PMC146525

[32] Greiner S , Lehwark P , Bock R . OrganellarGenomeDRAW (OGDRAW) version 1.3.1: expanded toolkit for the graphical visualization of organellar genomes. Nucleic Acids Res. 2019;47(W1):W59–64. 30949694 10.1093/nar/gkz238 PMC6602502

[33] Rice P , Longden I , Bleasby A . EMBOSS: the European molecular biology open software suite. Trends Genet. 2000;16:276–7. 10827456 10.1016/s0168-9525(00)02024-2

[34] Mayor C , Brudno M , Schwartz JR , Poliakov A , Rubin EM , Frazer KA , et al. VISTA: visualizing global DNA sequence alignments of arbitrary length. Bioinformatics. 2000;16:1046–7. 11159318 10.1093/bioinformatics/16.11.1046

[35] Dubchak I , Ryaboy DV . VISTA family of computational tools for comparative analysis of DNA sequences and whole genomes. Methods Mol Biol. 2006;338:69–89. 16888351 10.1385/1-59745-097-9:69

[36] Kurtz S , Schleiermacher C . REPuter: fast computation of maximal repeats in complete genomes. Bioinformatics. 1999;15:426–7. 10366664 10.1093/bioinformatics/15.5.426

[37] Beier S , Thiel T , Münch T , Scholz U , Mascher M . MISA‐web: a web server for microsatellite prediction. Bioinformatics. 2017;33:2583–5. 28398459 10.1093/bioinformatics/btx198 PMC5870701

[38] Katoh K , Kuma K , Miyata T , Toh H . Improvement in the accuracy of multiple sequence alignment program MAFFT. Genome Informatics. 2005;16:22–3. 16362903

[39] Tamura K , Stecher G , Peterson D , Filipski A , Kumar S . MEGA6: molecular evolutionary genetics analysis version 6.0. Mol Biol Evol. 2013;30:2725–9. 24132122 10.1093/molbev/mst197 PMC3840312

[40] Thompson JD , Gibson TJ , Higgins DG . Multiple sequence alignment using ClustalW and ClustalX. Curr Protoc Bioinformatics. 2002; Chapter 2:Unit 2.3. 10.1002/0471250953.bi0203s00 18792934

[41] Lalitha S . Primer premier 5. Biotech Softw Internet Rep. 2000;1:270–72.

[42] Tan W , Gao H , Zhang H , Yu X , Tian X , Jiang W , et al. The complete chloroplast genome of Chinese medicine (Psoralea corylifolia): Molecular structures, barcoding and phylogenetic analysis. Plant Gene. 2020;21:100216.

[43] Liu W , Kong H , Zhou J , Fritsch P , Hao G , Gong W . Complete chloroplast genome of *cercis chuniana* (Fabaceae) with structural and genetic comparison to six species in Caesalpinioideae. Int J Mol Sci. 2018;19:1286. 10.3390/ijms19051286 PMC5983592 29693617

[44] Dong W , Xu C , Cheng T , Lin K , Zhou S . Sequencing angiosperm plastid genomes made easy: a complete set of universal primers and a case study on the phylogeny of saxifragales. Genome Biol Evol. 2013;5:989–97. 23595020 10.1093/gbe/evt063 PMC3673619

[45] Xie DF , Yu Y , Deng YQ , Li J , Liu HY , Zhou SD , et al. Comparative analysis of the chloroplast genomes of the Chinese endemic genus *Urophysa* and their contribution to chloroplast phylogeny and adaptive evolution. Int JMol Sci. 2018;19:1847. 10.3390/ijms19071847 PMC6073864 29932433

[46] Sugita M , Sugiura M . Regulation of gene expression in chloroplasts of higher plants. Plant Mol Biol. 1996;32:315–26. 8980485 10.1007/BF00039388

[47] Jansen RK , Raubeson LA , Boore JL . Methods for obtaining and analyzing whole chloroplast genome sequences. Methods Enzymol. 2005;395:348. 15865976 10.1016/S0076-6879(05)95020-9

[48] Li Y , Zhou J , Chen X , Cui Y , Xu Z , Li Y , et al. Gene losses and partial deletion of small single‐copy regions of the chloroplast genomes of two hemiparasitic *Taxillus* species. Sci Rep. 2017;7:12834. 29026168 10.1038/s41598-017-13401-4 PMC5638910

[49] Chen D , Peng R , Li L , Zhang X , Wang Y . Analysis of genetic relationships of *Pueraria thomsonii* based on SRAP markers. J China J Chin Mater Med. 2011;36:538–41. 21657066

[50] Gietler M , Nykiel M , Orzechowski S , Fettke J , Zagdańska B . Proteomic analysis of *S*‐nitrosylated and *S*‐glutathionylated proteins in wheat seedlings with different dehydration tolerances. Plant Physiol Biochem. 2016;108:507–18. 27596017 10.1016/j.plaphy.2016.08.017

[51] Baxter RM , White VT , Zahid ND . The modification of the peptidyltransferase activity of 50‐S ribosomal subunits, LiCl‐split proteins and L16 ribosomal protein by pyridoxal phosphate. Eur J Biochem. 1980;110:161–6. 6254759 10.1111/j.1432-1033.1980.tb04851.x

[52] Castillo‐Davis CI , Mekhedov SL , Hartl DL , Koonin EV , Kondrashov FA . Selection for short introns in highly expressed genes. Nat Genet. 2002;31:415–8. 12134150 10.1038/ng940

[53] Prachumwat A , DeVincentis L , Palopoli MF . Intron size correlates positively with recombination rate in *Caenorhabditis elegans* . Genetics. 2004;166:1585–90. 15082572 10.1534/genetics.166.3.1585 PMC1470791

