# Supplementary figures and images for: Chloroplast genomes of two Pueraria DC. species: sequencing, comparative analysis and molecular marker development
Source: FEBS Open Bio. 2021 Dec 26;12(2):349–61. doi: 10.1002/2211-5463.13335 (PMC8804624; doi:10.1002/2211-5463.13335)

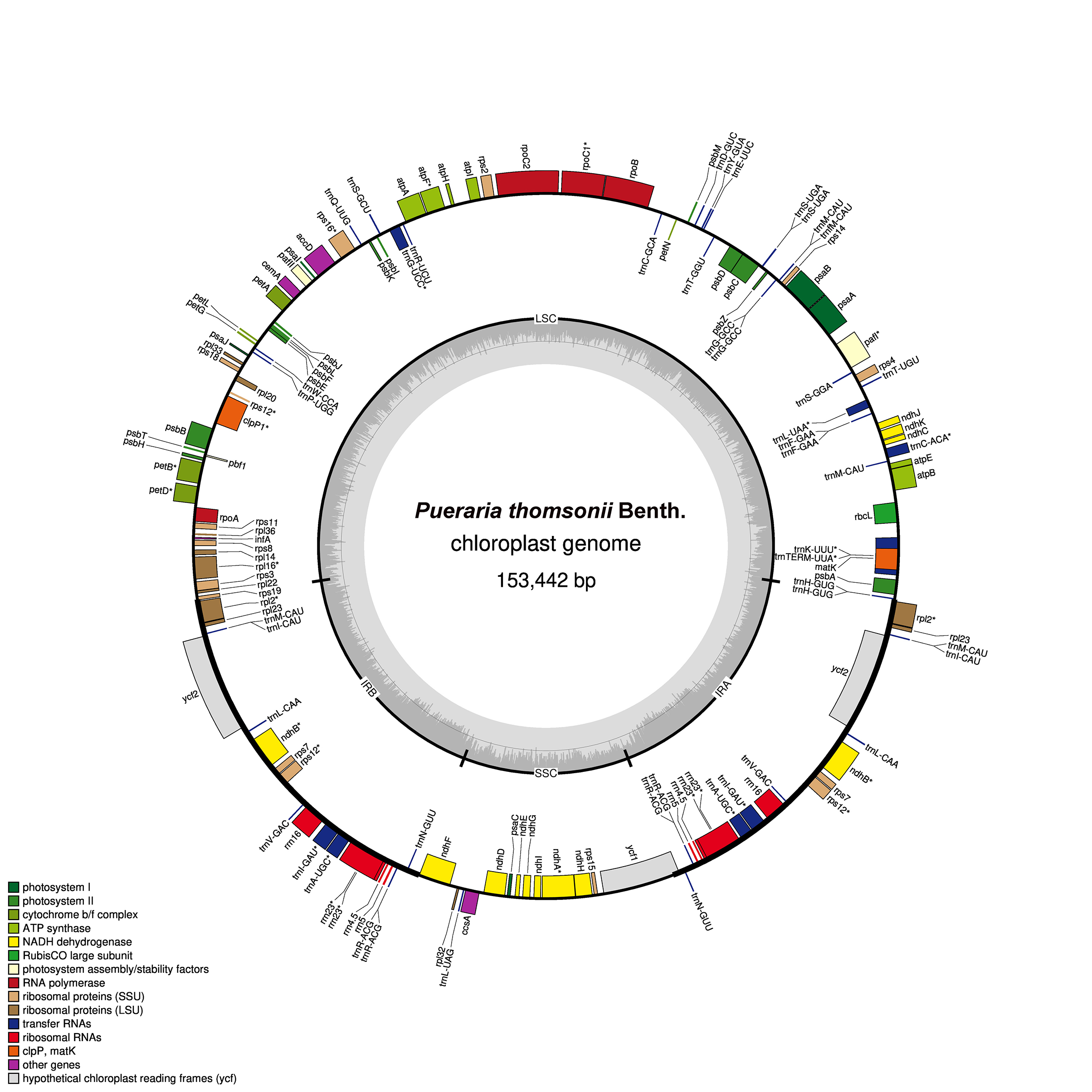

Supplement: Supplementary file 1 — Fig. S1. Circular Gene map of the complete CP genome of Puerariae thomsonii. Genes drawn inside the circle are the transcribed clockwise, and those on the outside are transcribed counter‐clockwise. The dark gray area in the inner circle corresponds to the GC content, whereas the light gray refers to AT content. LSC: large single copy region, SSC: small single copy region, IRa, IRb: copies of inverted repeat regions. [file FEB4-12-349-s002.jpg]
